# Effect of climatic oscillations on small pelagic fisheries and its economic profit in the Gulf of Cadiz

**DOI:** 10.1007/s00484-021-02223-9

**Published:** 2021-11-27

**Authors:** Jairo Castro-Gutiérrez, Remedios Cabrera-Castro, Ivone Alejandra Czerwinski, José Carlos Báez

**Affiliations:** 1 Departamento de Biología, Facultad de Ciencias del Mar y Ambientales, Universidad de Cádiz. Campus de Excelencia Internacional del Mar (CEIMAR), Avda. República Saharaui, s/n, 11510 Puerto Real, Cadiz Spain; 2 Instituto Universitario de Investigación Marina (INMAR), Campus de Excelencia Internacional del Mar (CEIMAR), Avda. República Saharaui, s/n, 11510 Puerto Real, Cadiz Spain; 3 Instituto Español de Oceanografía (IEO-CSIC), Centro Oceanográfico de Cadiz, Muelle de Levante, s/n, 11006 Puerto Pesquero, Cadiz Spain; 4 Instituto Español de Oceanografía (IEO-CSIC), Centro Oceanográfico de Málaga, Puerto Pesquero de Fuengirola s/n, 29640 Fuengirola, Spain; 5 Instituto Iberoamericano de Desarrollo Sostenible, Universidad Autónoma de Chile, Temuco, Chile

**Keywords:** Climate oscillations, Fisheries bioeconomy, Gulf of Cadiz, Small pelagic, Generalised linear models

## Abstract

Several studies have shown the effect of climatic oscillations on fisheries. Small pelagic fish are of special global economic importance and very sensitive to fluctuations in the physical environment in which they live. The main goal of this study was to explore the relationship between the North Atlantic Oscillation (NAO), the East Atlantic pattern (EA), and the Arctic Oscillation (AO) on the landings and first sale prices of the most representative small pelagic commercial species of the purse-seine fisheries in the Gulf of Cadiz (North East Atlantic), the European anchovy *Engraulis encrasicolus* and the European sardine *Sardine pilchardus*. Generalised linear models (GLMs) with different data transformations and distribution errors were generated to analyse these relationships. The best results of the models were obtained by applying a moving average of order 3 to the dataset with a double weighted median. Our results demonstrate relationships between NAO, AO, and EA and European anchovy and sardine landings. These cause an indirect effect on the first sale price in markets through catch variations, which affect the price according to the law of supply and demand. The limitations of this study and management implications are discussed.

**Supplementary Information:**

The online version contains supplementary material available at 10.1007/s00484-021-02223-9.

## Introduction

The effect of climatic variability of large-scale climatic phenomena on small pelagic fisheries is a well-described fact in scientific literature (for example Chávez et al. 2003; Alheit et al. 2009; Checkley et al. 2017; Báez et al. 2019a; Báez et al. 2021). Understanding the effect of these variations is essential for better management of fish stocks (Leitão et al. 2014; Leitão 2015), as the climatic variables that affect the ocean can alter feeding patterns, growth, and the migratory behaviour of the fisheries’ target species (Miller et al. 2010). Many authors have shown that climatic oscillations can affect the catchability of species (Rubio et al. 2015), their physical condition (Basilone et al. 2006; Báez et al. 2019b), and fishing effort (Rubio et al. 2015), amongst others (for example, see Báez et al. 2021 for a review). Moreover, recent studies have shown that climatic oscillation could explain first sale fish market prices (Fernández et al. 2020). The first sale price of a fishery product is the price that the first buyer in the market chain has paid for the product. This is established in Spain through public action at the fish market.

According to Barnston and Livezey (1987) and Wang et al. (2005), the three most important interannual sources of climatic variability patterns in the Northern Hemisphere that affect the Atlantic Ocean are North Atlantic Oscillation (NAO), East Atlantic pattern (EA), and Arctic Oscillation (AO).

The NAO is defined by the redistribution of atmospheric masses between the subtropical high surface pressure centre located near the Azores (Azores High) and the centre of low surface pressures near Iceland (Icelandic Low) (Hurrell and Deser 2009; Báez et al. 2021). The NAO phases determine the strength and direction of the westerly winds. When the pressures of the subtropical high and the polar low intensify, the NAO is in its positive phase. This event increases the number and intensity of the disturbances that cross the Atlantic towards north-western Europe, leading to hot and wet winters in north-western Europe, whilst decreasing rainfall in winter in the Iberian Peninsula. When the NAO is in its negative phase, the opposite happens (Hurrell 1995). Sánchez et al. (2007) showed how on a regional scale in the Gulf of Cadiz, the positive and negative phases of the NAO are associated with upwellings and downwellings, which in turn are related to temperature variations. The NAO shows both intra- and inter-annual variation (Hurrell 1995).

The EA consists of a North–South dipole that spans the North Atlantic Ocean, with centres near 55°N, 20–35°W and 25–35°N, 0–10°W. It is structurally similar to the NAO, and its anomaly centres are found to the southeast of it. A positive EA phase is associated with an increase in the mean rainfall in northern Europe and Scandinavia and a decrease in rainfall in southern Europe and is also associated with an increase in temperatures in the north of the Iberian Peninsula (Sáenz et al. 2001) and vice versa (Rodríguez-Puebla et al. 1998, 2001). The negative phase is associated with upwellings on the west coast of the Iberian Peninsula (De Castro et al. 2008).

The AO was described by Thompson and Wallace (1998) as the main dominant mode of variability in the Northern Hemisphere. It is the dominant pattern of non-seasonal variations in atmospheric pressure north of 20°N and is characterised by anomalies in pressure of positive or negative magnitudes in the Arctic and anomalies of opposite magnitudes located between 37 and 45°N. The positive phase is characterised by a strengthening of the polar vortex from the surface to the lower stratosphere, resulting in storms in the North Atlantic and a shift of droughts to the Mediterranean. During the negative phase, the continental cold air spreads through western Europe whilst the storms intensify in the Mediterranean region (Ambaum et al. 2001; Rodríguez-Puebla et al. 2002). Different studies have shown that there is a connection between the NAO and the AO (Overland et al. 2010; Báez et al. 2013a). During winter, both AO and NAO tend to be in a positive phase when the stratospheric vortex is strong (Douville 2009).

The Gulf of Cadiz is located to the southwest of the Iberian Peninsula and, due to its geographical position, presents a continuous exchange of water masses between the Atlantic Ocean and the Mediterranean Sea through the Strait of Gibraltar, which makes it a highly dynamic area (Vargas et al. 2002). The mouths of the important rivers such as the Guadalquivir or the Guadiana, amongst others, turn the waters around them into very productive areas through a major contribution in nutrients (Uriarte et al. 1996; Pérez-Rubín et al. 1997; García-Lafuente and Ruiz 2007). Together with other physical–chemical and ecological processes, this contributes to making the Gulf of Cadiz a spawning, breeding, and juvenile area for many important fishery species such as the European anchovy *Engraulis encrasicolus* (Linnaeus, 1758) and the European sardine *Sardina pilchardus* (Walbaum, 1792) (Pérez-Rubín et al. 1997, 1999; García-Isarch et al. 2003a, b; Baldó et al. 2006).

In general, small pelagic fishes are a group of special economic importance for all countries. However, due to the peculiar hydrological and hydrodynamic processes in the Gulf of Cadiz, small pelagic fisheries represent an important economic resource (Baldó et al. 2006). The fishing resources of this area are characterised by the seasonal alternation of various types of fishing gear with different target species, which are related to the variation in abundance of the species in accordance with the season. These variations are affected by both biological factors (dependent on the ecology of the species) and economic factors (dependent on the market value) (Ruíz et al. 2017). In the purse-seine fleet of the Gulf of Cadiz, the main target species is the European anchovy that inhabits these waters due to its high first sale price, but it also captures other species of great fishing interest, mainly European sardine (the second most important species caught by purse seiners from the Gulf of Cádiz) (Millán 1992; Casimiro-Soriguer et al. 2000; Ruíz et al. 2017; ICES 2020). Since 2002, the low recruitment of the European anchovy has kept the population at historical critical levels (ICES 2008). Over the last 15 years, the Iberian Peninsula European sardine stock decreased severely due to prolonged low recruitment and high catch levels, with serious social and economic impacts on the Portuguese and Spanish purse seine fisheries (Silva et al. 2019). After a last good recruitment in 2004, during the years 2006 to 2010, European sardine recruitment decreased to very low levels affecting catches in subsequent years (ICES 2011; Garrido et al. 2017).

The Spanish fleet includes approximately 87 vessels with a length range between 10 and 25 m (Ruíz et al. 2017). From 2002 to 2004, the large tonnage purse seine fleet returned to fishing. However, to counterbalance this increase in fishing effort, authorities used a combination of fishery closures and reductions in the number of purse-seine vessels (ICES 2007). The Gulf of Cadiz purse-seine fleet mainly operates from the home-base harbours of Barbate, Punta Umbría, and Isla Cristina. Vessels from Barbate harbour are relatively large and until 1999 could also fish in Moroccan waters outside of the EU Economic Exclusive Zone (EEZ) (the agreement between Morocco and the EU was terminated on 30 November 1999 and was not renewed again until 2006) (García del Hoyo et al. 2004; García-Isarch et al. 2012). These conditions along with environmental pressures make landings from European anchovy and European sardine notably fluctuate jeopardising the biological stability of the species and its economic sustainability, risking the jobs of nearly 800 employees (Ruíz et al. 2017).

The main aim of this present study is to determine the relationship between the most important interannual climatic oscillations in the Northern Hemisphere (i.e., climatic oscillations NAO, AO, and EA) and the European anchovy and sardine landings, and first sale prices in the Gulf of Cádiz. In the same way, this study tries to use the climatic oscillations as a proxy to perform ecological predictions, to establish the conceptual bases of the relationships, and to know and anticipate the fisheries’ level of resilience to climate oscillation variability.

## Material and methods

### Study area

**Fig. 1:**
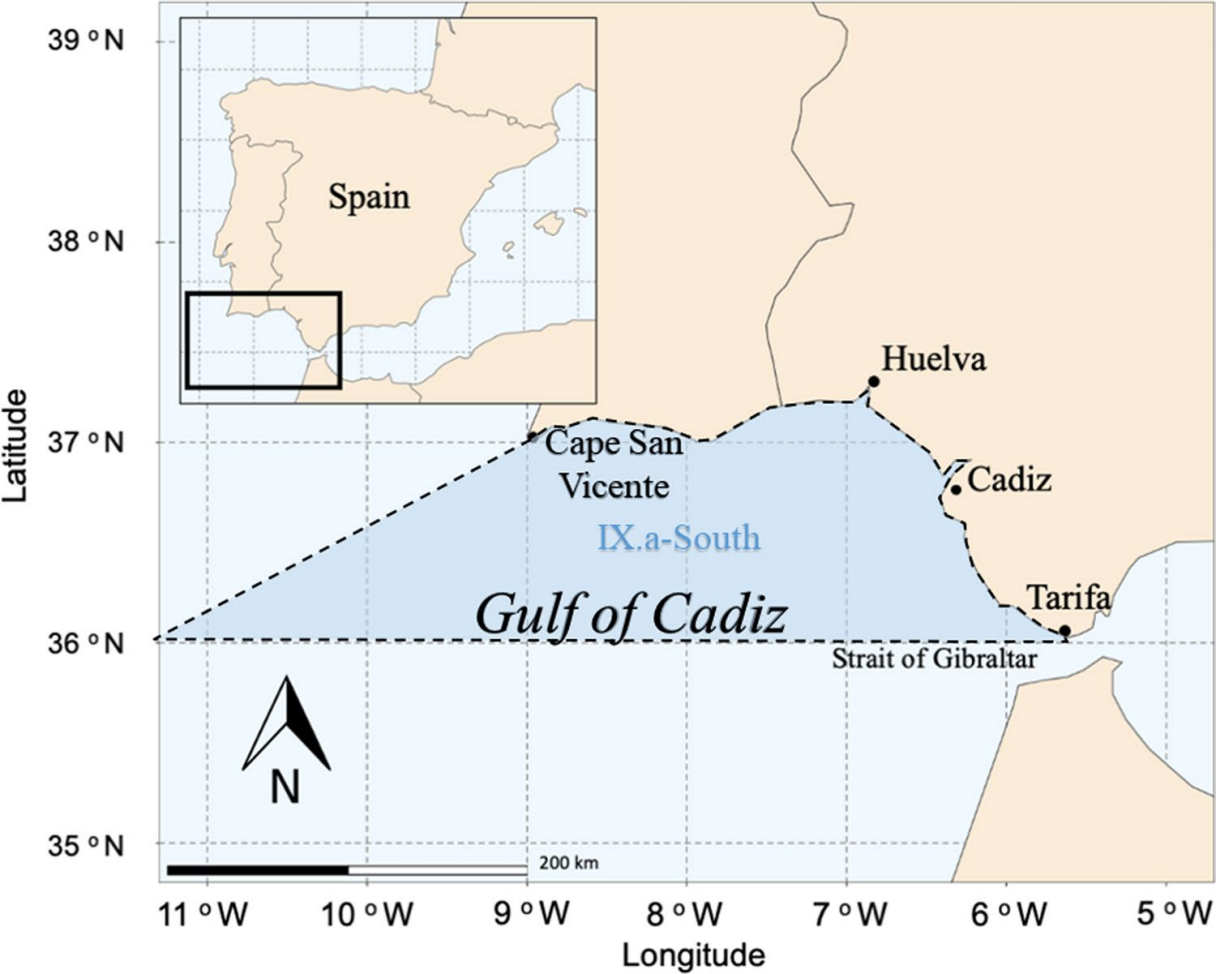
Gulf of Cadiz (ICES subdivision IX.a-South, Southwest Spain)

For this study, the Gulf of Cadiz has been limited to the waters between the meridian of Punta Marroquí (near Tarifa) and Cape San Vicente, covering approximately 300 km of coastline (Bellido et al. 2000). The study area is included in the ICES subdivision IX.a-South (Fig. 1) which is the area where the purse seine fleet fish.

### Fisheries data

European anchovy and European sardine landings’ data has been used as a proxy for species abundance (García et al. 2003). Annual landings data (t) and first sale prices (€/kg) of the species from 1985 to 2017 were extracted from the Fisheries Information System Database of the Junta de Andalucía (Andalusia Regional Government) (Sistema de Información Andaluz de Comercialización y Producción Pesquera of the Junta de Andalucía 2020). The first sale prices were standardised with the annual Consumer Price Index (CPI) provided by the Spanish National Institute of Statistics (Instituto Nacional de Estadística, 2020) performing a restatement of stockholders’ equity (Fernández et al. 2020). Hereinafter, this variable is named as standard first sale price.

### Atmospheric oscillation data

Three climate indices have been selected: North Atlantic Oscillation (NAO), Arctic Oscillation (AO), and the East Atlantic pattern (EA). Monthly NAO, AO, and EA indices values were taken from the website of the National Oceanic and Atmospheric Administration (NOAA): https://www.noaa.gov/. Six variables were extracted from each climatic index: annual index (average of all months of the year), winter index (average for the months December to March), summer index (average for the months June to August), and 3 lagged indices (using different lags to compare fishing data from one year with climatic indices from previous years, in order to analyse the effect on the early life stages and the influence on recruitment). Up to a 3-year lag between the influence of environmental conditions and anchovy landings was used based on the age classes that were captured the most in the study area (ICES 2020) which could suggest a knock-on effect from the environmental oscillations. Each variable was also square or cube transformed. The use of variables with quadratical and cubical transformations could show potentially, ecologically meaningful relationships with landings. The database was finally composed of 108 climate variables. The synopsis of the variables and sub-variables used in this study is summarised in Table 1.

**Table 1:** Summary of the variables used in this study. The letters “w” and “s” after the name of the climatic variables correspond to the winter and summer sub-variables, respectively. The number after the name of the climatic variables means the amount of lag (in years) used. All variables and sub-variables were squared (_sq) and cubed (_cb)

Climatic oscillations	Original variables	Transformed variables						
North Atlantic Oscillation (NAO)	NAO	NAO_sq	NAO1	NAO1_sq	NAO2	NAO2_sq	NAO3	NAO3_sq
		NAO_cb		NAO1_cb		NAO2_cb		NAO3_cb
	NAOw	NAOw_sq	NAOw1	NAOw1_sq	NAOw2	NAOw2_sq	NAOw3	NAOw3_sq
		NAOw_cb		NAOw1_cb		NAOw2_cb		NAOw3_cb
	NAOs	NAOs_sq	NAOs1	NAOs1_sq	NAOs2	NAOs2_sq	NAOs3	NAOs3_sq
		NAOs_cb		NAOs1_cb		NAOs2_cb		NAOs3_cb
Arctic Oscillation (AO)	AO	AO_sq	AO1	AO1_sq	AO2	AO2_sq	AO3	AO3_sq
		AO_cb		AO1_cb		AO2_cb		AO3_cb
	AOw	AOw_sq	AOw1	AOw1_sq	AOw2	AOw2_sq	AOw3	AOw3_sq
		AOw_cb		AOw1_cb		AOw2_cb		AOw3_cb
	AOs	AOs_sq	AOs1	AOs1_sq	AOs2	AOs2_sq	AOs3	AOs3_sq
		AOs_cb		AOs1_cb		AOs2_cb		AOs3_cb
East Atlantic pattern (EA)	EA	EA_sq	EA1	EA1_sq	EA2	EA2_sq	EA3	EA3_sq
		EA_cb		EA1_cb		EA2_cb		EA3_cb
	EAw	EAw_sq	EAw1	EAw1_sq	EAw2	EAw2_sq	EAw3	EAw3_sq
		EAw_cb		EAw1_cb		EAw2_cb		EAw3_cb
	EAs	EAs_sq	EAs1	EAs1_sq	EAs2	EAs2_sq	EAs3	EAs3_sq
		EAs_cb		EAs1_cb		EAs2_cb		EAs3_cb

### Generalised linear models (GLM)

Two types of GLM (linear and logistic) were performed with 7 different transformations of the data, obtaining 7 different smoothing methods (Table 2). Data smoothing is a statistical technique that consists of removing outliers from a data set to make a pattern more visible. This improves the fit of the residuals to the family of GLMs and removes noise from the data (Tong 1976).

**Table 2:** Description of the different data transformations performed in the present study

Model	Transformation
Raw	Raw dataset without transformation
MA_all	Moving average of order 3 applied to all variables
MA_resp	Moving average of order 3 applied only to the response variable
WMA_all	Moving average of order 3 where the central value has twice the weight applied to all variables
WMA_resp	Moving average of order 3 where the central value has twice the weight applied only to the response variable
Logit_mean	Binary transformation of the response variable according to whether the value of the observation was higher (1) or lower (0) than the total mean
Logit_cp	Binary transformation of the response variable according to whether the value is higher (1) or lower (0) than the mean in the corresponding changepoint period

Linear GLMs were performed using the gamma error distribution for continuous variables, and logistic regressions were performed using the binary error distribution for response variables composed of 1 s and 0 s (Zuur et al. 2009).

First, GLM models were generated using landings (in tons, t) as the response variable and the climatic indices as explanatory variables. To find the explanatory variables that best fit each model, the step forward methodology was used, carried out as follows: (1) A battery of models were created with the response variable and with each of the climate oscillations as explanatory variables; (2) the model with the lowest AICc and the highest percentage of deviance explained was selected; and (3) if any of the explanatory variables were not introduced in a significant way, they were eliminated from the model.

To create bioeconomic models, we used the ecological variables selected in the previous GLM models. In this way, ecological interpretations can be made from the bioeconomic models, and therefore ecological management can be linked to economical implications. The bioeconomic models were fitted using the same error distribution and link as the ecological model. A stepwise backward selection (using the “step” function) was performed. This method iteratively removes the least contributive predictors and stops when it has found the most parsimonious model (Crawley 2012).

In Online Resource 1, we provide a detailed explanation on data analysis, bioeconomic models, and model evaluation. All statistical models were generated with R version 4.0 and RStudio version 1.1.463 (RStudio Team 2016; R Core Team 2020).

## Results

A preliminary exploration was performed for each species (Online Resource 2). A total of 1520 GLMs were performed for both species. The best results of the linear regression models are shown in Table 3.

**Table 3:** Synopsis of the ecological and bioeconomic linear GLMs for European anchovy and European sardine. The letters “w” and “s” after the name of the climatic variables correspond to the winter and summer sub-variables, respectively. The number after the name of the climatic variables means the amount of lag (in years) used. Suffix “_sq” and “_cb” means square and cube transformation, respectively

Data transformation (model type)	Species	Explanatory variables	Deviance explained (%)	VIF	AC	*R*^2^ observed vs predicted
Raw	Anchovy	NAOs2 *	38.09	1.18	No	0.32
(ecological)		AOw1 *		1.13		
		EA3_sq #		1.17		
Raw	Sardine	NAOw1 **	40.16	1.11	No	0.35
(ecological)		AO3_cb **		1.14		
		EA_cb *		1.04		
Raw	Anchovy	Anchovy landings (t) #	22.90	1.08	Yes	0.17
(bioeconomic)		NAOs2 *		1.08		
MA_all	Anchovy	NAOw3_cb ***	75.99	1.53	No	0.75
(ecological)		AO3 ***		1.16		
		EA3 ***		1.35		
MA_all	Sardine	NAOw1 ***	67.92	1.20	No	0.69
(ecological)		AOs1 ***		1.65		
		EAs2 **		1.43		
MA_all	Anchovy	Anchovy landings (t) **	47.70	1.41	Yes	0.37
(bioeconomic)		AO3 *		1.44		
		EA3 ***		1.56		
MA_resp	Anchovy	NAOw3_sq **	51.19	1.05	Yes	0.56
(ecological)		AO2 *		1.02		
		EA3 **		1.07		
MA_resp	Sardine	AO *	43.78	1.00	Yes	0.33
(ecological)		EA_sq ***		1.00		
WMA_all	Anchovy	Oasis ***	80.23	1.09	No	0.80
(ecological)		EAs3_cb ***		1.09		
WMA_all	Sardine	NAOw1 ***	75.31	1.59	No	0.70
(ecological)		AOs1_cb ***		2.51		
		EAw3_sq ***		1.88		
WMA_all	Anchovy	Anchovy landings (t) ***	65.09	2.09	Yes	0.59
(bioeconomic)		AOs_sq *		1.41		
		EAs3_cb ***		1.80		
WMA_resp	Anchovy	NAOw3_sq **	49.53	1.06	Yes	0.53
(ecological)		AO2 *		1.03		
		EA3 **		1.08		
WMA_resp	Sardine	NAOw1 *	43.98	1.34	Yes	0.35
(ecological)		AO3_cb *		1.04		
		EA_cb **		1.35		

Asterisks indicate a significance level of 0 (***), 0.001 (**), 0.01 (*), and 0.05 (#)

*VIF* variance inflation factor, *AC* autocorrelation, *NAO* North Atlantic Oscillation, *AO* Arctic Oscillation, *EA* East Atlantic pattern

The best results of the European anchovy ecological GLMs were obtained using the gamma error structure and the inverse link function. The three climatic variables were significant in all of the ecological models, except WMA_all (i.e., the transformation moving average of order 3 where the median of all variables is double weighted), which only included the variables AO and EA. The variable EA was introduced 3-year lagged into all of the ecological models and, except for the WMA_all model, the NAO variable was introduced into all of the linear ecological GLMs. In most models, NAO was introduced as the sub-variable NAO winter with a 3-year lag. The results showed an increase in the deviance and goodness of fit when both the response and explanatory variables are smoothed. The WMA_all and MA_all models (i.e., the transformation moving average of order 3 applied to all variables) explained the highest deviance percentages (~ 80 and ~ 76%, respectively). Multicollinearity was not found in any ecological model (VIF < 10). The Raw (without data transformation), MA_all, and WMA_all models did not present autocorrelation in the residuals. The ecological models that best adjusted to the data were the WMA_all and MA_all models, with an observed vs predicted *R*^2^ of 0.804 and 0.752, respectively.

For European sardine, all linear GLMs were better fitted using the identity link function, except for the Raw model that used the inverse link function. The three climatic indices were introduced significantly in all the models except in the MA_resp (i.e., the transformation moving average of order 3 applied only to the response variables), which only included the variables AO and EA. The NAO climatic index was always entered as the sub-variable NAOw1, corresponding to the winter NAO with 1-year lag. As in the European anchovy models, smoothing all variables in the models (_all) improved the explained deviance and goodness of fit. The WMA_all model explained the highest percentage of deviance (~ 75%) followed by the MA_all model (~ 68%). The model with the lowest percentage of explained deviance was Raw with 40.16%. No model presented multicollinearity. The Raw, MA_all, and WMA_all models did not show residual autocorrelation. The model that best fit the data was WMA_all (*R*^2^ = 0.703) followed by the MA_all model (*R*^2^ = 0.685).

The best results of the logistic GLMs are shown in Table 4.

**Table 4:** Synopsis of the ecological logistic GLMs for European anchovy and European sardine. The letters “w” and “s” after the name of the climatic variables correspond to the winter and summer sub-variables, respectively. The number after the name of the climatic variables means the amount of lag (in years) used

Data transformation	Species	Explanatory variables	Deviance explained (%)	VIF	AC	AUC	Accuracy (%)	Precision (%)
Logit_mean	Anchovy	AOs3 *	48.44	2.02	No	0.921	36.36	75.00
		EAw2 *		2.02				
Logit_mean	Sardine	NAO2_sq #	55.13	1.04	No	0.932	42.42	85.71
		EAs_sq #		1.04				
Logit_cp	Anchovy	EA3 **	22.66	–	No	0.805	54.54	66.67
Logit_cp	Sardine	NAOs2 *	51.66	1.46	No	0.912	45.45	93.33
		AOw1_sq *		1.40				
		EAs3_sq *		1.22				

Asterisks indicate a significance level of 0.001 (**), 0.01 (*), and 0.05 (#)

*VIF* variance inflation factor, *AC* autocorrelation, *AUC* area under the curve, *AO* Arctic Oscillation, *EA* East Atlantic pattern

For European anchovy, the logistic models significantly introduced the variable EA but not the variable NAO. The variable AO was only introduced in the Logit_mean model (i.e., binary transformation of the response variable according to whether the value is higher (1) or lower (0) than the mean). The Logit_mean model explained 48.44% of the deviance against the 22.66% of the Logit_cp model (i.e., binary transformation of the response variable according to whether the value of the observation was higher (1) or lower (0) than the mean in the corresponding changepoint period). The Logit_mean model did not present multicollinearity amongst variables. The residuals of the models showed not to be autocorrelated. The logistic models showed to be well fitted according to the Hosmer–Lemeshow statistic (*p* > 0.05), though the AUC value of the Logit_mean model was higher than that of Logit_cp, showing the model had a better fit. The Logit_mean model obtained lower accuracy (~ 36%) and higher precision (75%) than the Logit_cp model (~ 55 and ~ 67%, respectively).

For European sardine, the variables NAO and EA were introduced in both logistic GLMs, whilst the variable AO was only introduced in the Logit_cp (i.e., binary transformation of the response variable according to whether the value is higher (1) or lower (0) than the mean in the corresponding changepoint period) model. The Logit_mean model (i.e., binary transformation of the response variable according to whether the value of the observation was higher (1) or lower (0) than the total mean) presented a higher percentage of explained deviance (~ 55%) than the Logit_cp model (~ 52%). The residuals of the models showed not to be autocorrelated. The models showed to be well fitted according to the Hosmer–Lemeshow statistic (*p* > 0.05), though the AUC value of the Logit_mean model was higher than that of Logit_cp, showing a better fit. The Logit_mean model obtained lower accuracy (~ 42%) and precision (~ 86%) than the Logit_cp model (~ 45 and ~ 94%, respectively).

**Fig. 2:**
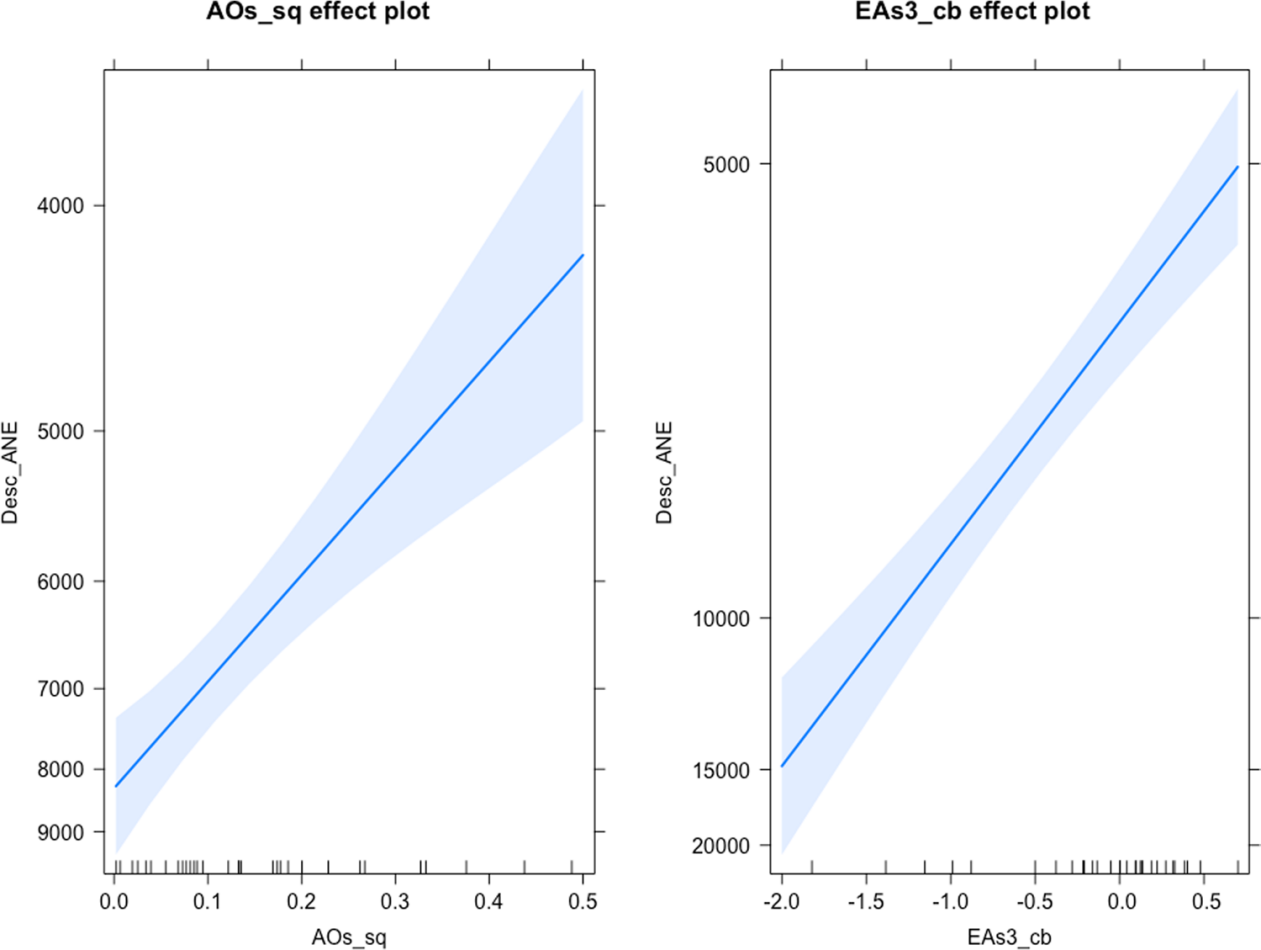
Partial effects plots of the European anchovy WMA_all model (Moving average of order 3 where the median of all the variables is double weighted). Key: Desc_ANE, European anchovy landings; AOs_sq, summer Arctic Oscillation squared; EAs3_cb, summer East Atlantic pattern with 3-year lag and cubed

**Fig. 3:**
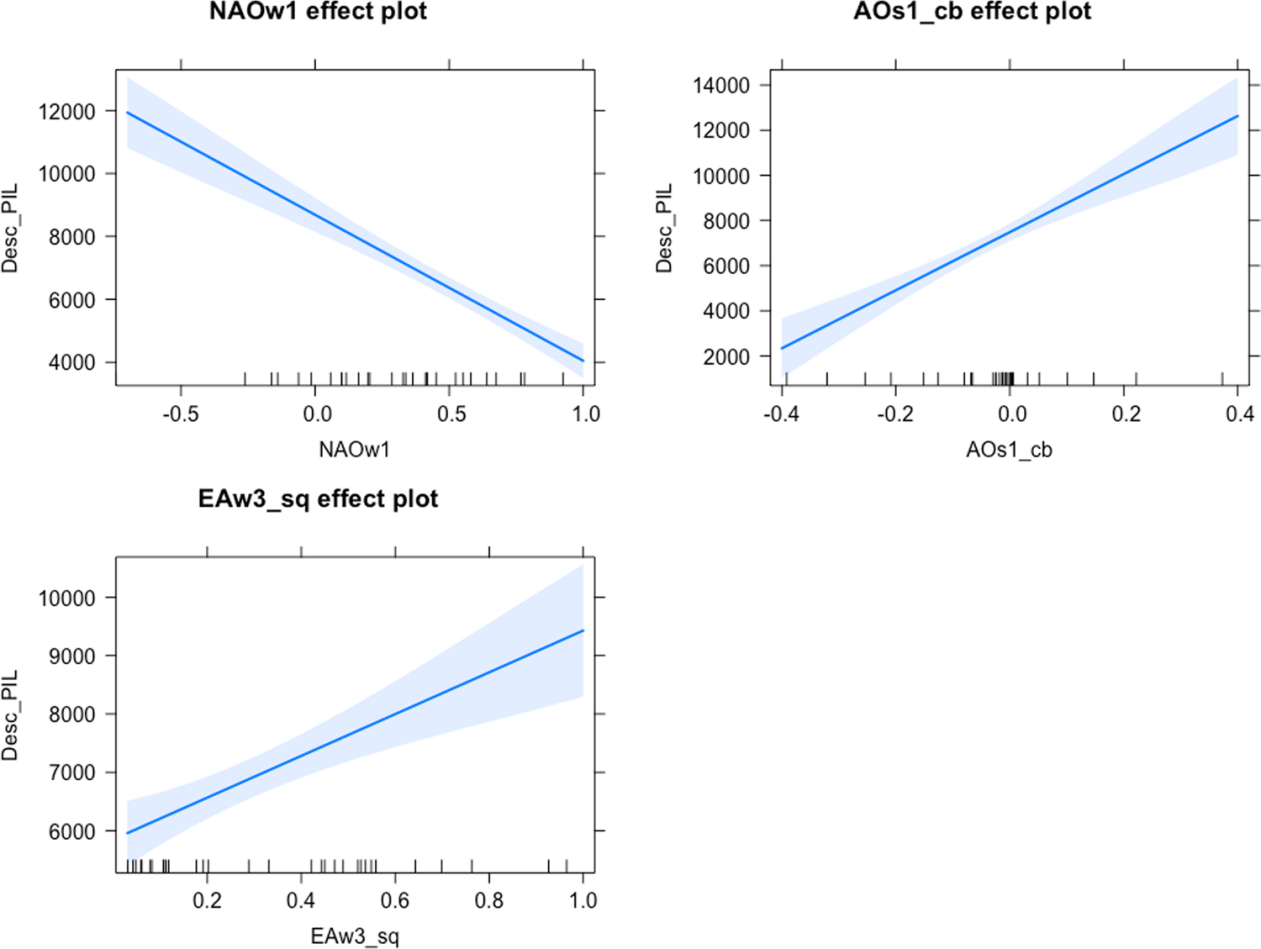
Partial effects of the European sardine WMA_all model (Moving average of order 3 where the median of all variables is double weighted). Key: Desc_PIL, European sardine landings; NAOw1, winter North Atlantic Oscillation with 1-year lag; AOs1_cb, summer Arctic Oscillation with 1-year lag and cubed; EAw3_sq, winter East Atlantic pattern with 3-year lag and squared

Models that presented an explained deviance of less than 50% were discarded. Finally, after comparing the amount of deviance explained by the model and the goodness-of-fit, the model that best fits both species’ landings is the WMA_all model, which corresponds to the transformation of the data by means of a moving average whose median is double weighted. For European anchovy, the partial effects plot (Fig. 2) shows that negative values of the EA variable 3 years prior to the landings positively affect the response variable (and vice versa) and extreme values (both positive and negative) of AO during the summer affects landings of the same year negatively. For European sardine, partial effects plot (Fig. 3) shows how negative values of the NAO variable and positive AO and EA values have a positive effect on European sardine landings.

### Bioeconomic models

In a first step, in the case of the European anchovy, residual autocorrelation was found in all bioeconomic models performed. The bioeconomic model that best fit the data was WMA_all. Similarly, in the case of the European sardine, the step function did not return any bioeconomic model with significant explanatory variables, so no valid bioeconomic model could be generated.

For this reason, in a second step to improve and fix the assumptions of the bioeconomic models, these were repeated (using the same methodology as for the ecological ones) with two modifications: (1) splitting the dataset into two parts (1985–1999 and 2000–2017) and (2) including landings and first sale prices of the other species as potential explanatory variables. The hope was that by splitting the series after the year with the highest catch landings (and therefore the highest supply), the variability within each data subset would be smoothed. Furthermore, landings were included as explanatory variables as according to García del Hoyo (1997) the European sardine fishery is less economically profitable than the European anchovy; therefore, their landings could be negatively dependent on the European anchovy fishery.

The resulting models describing first sale prices had significant explanatory variables and no autocorrelation; these results can be found in Table 5.

**Table 5:** Results of the bioeconomic models for European anchovy and European sardine after the modifications: (1) splitting the dataset into two parts (1985–1999 and 2000–2017) and (2) including landings and first sale prices of the other species as potential explanatory variables

Response variable (period)	Explanatory variables	Deviance explained (%)	VIF	AC	*R*^2^ observed vs predicted
Anchovy first sale prices	Sardine prices ***	85.51	1.24	No	0.87
(1985–1999)	EAs3_cb *		1.24		
Anchovy first sale prices	Anchovy landings **	56.54	1.11	No	0.52
(2000–2017)	Sardine landings *		1.11		
Sardine first sale prices	Anchovy prices ***	82.50	1.20	No	0.83
(1985–1999)	Anchovy landings #		1.20		
Sardine first sale prices (2000–2017)	Sardine landings *	23.57	-	No	0.70

Asterisks indicate a significance level of 0 (***), 0.001 (**), 0.01 (*), and 0.05 (#)

*EAs3_cb* summer East Atlantic pattern with 3-year lag and cubed, *VIF* variance inflation factor, *AC* autocorrelation

## Discussion

### Ecological models

In the case of European anchovy landings, we found that negative values of the EA variable 3 years prior to the landings would positively affect the response variable, and vice versa. In addition, extreme values (both positive and negative) of AO during the summer seem to negatively affect the landings of the same year (Fig. 2).

The summer East Atlantic pattern is the second dominant mode of summer low-frequency variability in the Euro-Atlantic region. A positive EA phase is associated with an increase in the mean rainfall in northern Europe and Scandinavia, and a decrease in rainfall in southern Europe and is also associated with an increase in temperatures in the north of the Iberian Peninsula (Sáenz et al. 2001) and vice versa (Rodríguez-Puebla et al. 1998, 2002). The onset of the European anchovy spawning period in the Gulf of Cadiz is related to the seasonal warming of the sea surface waters and the beginning of the stratification of the water column (Palomera 1992; García and Palomera 1996; Motos et al. 1996; Kideys et al. 1999; Millán 1999; Baldó et al. 2006). However, the biological characteristics of the small pelagic fishes make them highly sensitive to environmental forcing and extremely variable in their abundance (Alheit et al. 2009, 2012). In recent years, an increase in abundance and a gradual expansion northward of Round sardinella (*Sardinella aurita*) have been documented along the western Mediterranean coasts in relation to a progressive increase in sea water temperature (Sabatés et al. 2006, 2013; Tsikliras 2008); this species has been also found in the Gulf of Cadiz (Pérez-Rubín and Mafalda 2004). Round sardinella is a thermophilic species particularly frequent in the warmer waters of the eastern and southwestern Mediterranean basins (Sabatés et al. 2013). The Round sardine reproduces in summer, from late June to September, when surface waters reach the highest temperature of the year (Palomera and Sabatés 1990; Somarakis et al. 2002). Spawning periods of the European anchovy and Round sardinella coincide during the summer, and their temperature-related cohabitation has already been documented (e.g., Palomera and Sabatés 1990; Maynou et al. 2008; Zarrad et al. 2012; Karachle and Stergiou 2013; Diankha et al. 2015). Their cohabitation has also been demonstrated specifically in our study area, the Gulf of Cadiz (Perez-Rubín and Mafalda 2004). The partial spatial coincidence and the same vertical distribution of larvae, as well as their morphological similarity, may result in competition for food, as has been shown by other authors (Morote et al. 2008; Schismenou et al. 2008; Macías et al. 2014; Maynou et al. 2014; Albo-Puigserver et al. 2019; Effrosynidis et al. 2020). The numerical abundance of European anchovy versus Round sardinella has also been shown to be one of the reasons for displacement between species, so different temperature windows caused by different climatic indices may favour the presence of one species or another (Palomera and Sabatés 1990; Raab et al. 2013; Diankha et al. 2015). Mellado-Cano et al. (2019) demonstrated how the different phases of the EA regulate extreme high temperature events led by the NAO, even reversing the patterns of climate variability. The consideration of the EA as a temperature regulator together with the NAO can explain over 50% of the variation in temperature and can cause differences of up to 2 and 3 °C (Moore and Renfrew 2011). Therefore, we conclude that the inhibition of extremely warm temperatures by the EA in its negative phase could benefit the European anchovy by displacing a competitor, the Round sardinella. This results in a favourable spawning period for European anchovy, which has a knock-on effect that is reflected in the catches 3 years later.

On the other hand, the AO values that are affecting European anchovy landings are occurring without a time lag. Therefore, the AO is not having an effect on biomass, but on fishing effort. The AO values play an important role in determining extreme conditions such as frozen precipitations, strong winds, and extreme weather events in the Gulf of Cadiz (Rangel-Buitrago and Anfuso 2012; Cabrero et al. 2019). This suggests that extreme AO values result in the reduction of catch per unit effort and fishing effort due to adverse weather conditions.

In the case of European sardine landings, our results show that negative NAO during the previous winter and positive AO during the previous summer favour European sardine landings.

The negative phases of NAO induce major precipitation in southern Europe (Trigo et al. 2002, 2004; Hurrell et al. 2003; Vicente-Serrano et al. 2011; Báez et al. 2013a). Survival of European sardine larvae is closely related to vertical mixing and, consequently, to wind stress as a contributing mechanism (Lloret et al. 2004). Atmospheric disturbances affect marine sedimentation by a transfer of energy from the air to the sea, and from it to the seabed (De Luque 2008). This energy agitates the waters favouring the mixing of deep and superficial waters, increasing the contribution of nutrients to the surface (Báez et al. 2013b), affecting primary production positively, and this in turn affects the abundance of European sardine (Vargas-Yáñez et al. 2020). The increase in precipitation by the negative NAO also leads to an increase in runoff from the Iberian Peninsula (Trigo et al. 2004; Báez et al. 2013a). The fertilisation and local planktonic production by these plumes of continental freshwater support the growth and survival of the fish larvae of this species and avoid starvation (Chícharo et al. 2003; Santos et al. 2007). The Guadalquivir estuary (located at the mouth of the Guadalquivir and Guadiana rivers) is considered an important nursery area for many different species (Baldó et al. 2006). Therefore, the rainfall regime and the flow of the rivers, driven in turn by negative NAO phases, could be of great importance on a regional scale (Trigo et al. 2004). Moreover, Guisande et al. (2001) indicate the advantageous effect of the NAO in its negative phase on the recruitment of the European sardine due to the fact that the prevailing winds from the south drive the flow of water from the sea to the coast avoiding larval drift offshore, as well as the nutritional benefit from the mixture of nutrients in the column caused by a negative NAO phase.

Positive phases of the summer AO produce warmer conditions in the Iberian Peninsula, increasing dry winds (Marshal et al. 2001; Hall et al. 2014; Baldwin et al. 2007). Within the Atlantic Iberian waters, European sardine grows and improves in condition during spring and summer when temperature is close to the annual maxima and plankton production is high (Silva et al. 2008). Thus, the hydrographical conditions derived from a positive AO during the previous summer favour the biological conditions of the spawners during the following winter.

The climatic variable EA has been included as the same sub-variable previously introduced in the European anchovy model but here it was squared. The fact that it is squared implies that extreme values of the winter EA 3 years prior to the catches are beneficial to European sardine landings (Fig. 3). European sardine recruitment is greatly impaired when the temperature tends to be above or below the optimal range (Garrido et al. 2017). The impact of temperature on European sardine landings lead by the AO has been documented (Báez et al. 2019a). Therefore, we suggest that the positive effect of the variable EAw3 on European sardine landings is because this variable in its extreme values may be detrimental to European anchovy, with the consequent redirection of fishing effort from European anchovy to European sardine as the second main target species.

### Bioeconomic models

Our results show that the prices of European anchovy during the first sale stage are conditional on the price of European sardine and the climatic subvariate EA 3-year lag. At the same stage, the first sale price of European sardine depends on the prices and landings of European anchovy. The results of these models present a high amount of explained variance (> 80%) and a high fit (*R*^2^ predicted vs. adjusted > 0.80) which means that the resulting models can be considered as valid as well as good results. The inclusion of the EA variable in the model increases the percentage of explained variance by 9%, thus confirming the indirect effect of climate on the price of European anchovy. García del Hoyo (1997) found that the price of European anchovy during this period was sufficiently elastic to not depend on landings. Casimiro-Soriguer et al. (2000) studied the price of European sardine and anchovy during this period obtaining similar results in a similar period for European sardine first sale prices, depending only on his own landings.

During the second period (2000–2017), the results show that the price of European anchovy is dependent on its own landings and that of European sardine. During this period the European sardine prices are dependent only on its own landings. The results for European anchovy are considered acceptable although they have lower explained deviance and fit (~ 50%). These results confirm the relationship between European anchovy landings-standard first sale prices-climatic oscillations during both periods. The results in the European sardine bioeconomic model are considered insufficient to determine the factors modulating European sardine prices during the second period. There are very influential factors in the price of European sardine such as the demand by the canning industry or the high seasonal nature of the demand for its fresh consumption, especially during the summer months, where it can triple in value (Casimiro-Soriguer et al. 2000). Prices are not only dependent on the law of supply and demand but are also conditioned by multiple factors (biological, social, economic, institutional, commercial factors, etc.).

### Limitations of this study

Fishing effort is not available for the study period. Thereby, the catch per unit of effort could not be used as a proxy for stock abundance. However, given that the fleet in the fishing area has not changed greatly during the study period (ICES 2018), standardisation by unit effort may be dispensable (see OR3). Furthermore, landings have been used in other similar studies (García et al. 2003; Báez and Real 2011; Keller et al. 2014).

The patterns and processes reflected by climate indices are still unclear and difficult to discover (Straile and Stenseth 2007). Nevertheless, using global climate indices such as NAO, AO, or EA, the biological effects may exhibit a longer delay than with respect to any single local climate variables independently, which makes it possible for ecologists to anticipate them and make predictions (Báez et al. 2021). The mechanisms by which these climate indices act remain unclear, although there are well-established plausible mechanisms in the literature that could explain the results found, as detailed above.

Climatic oscillations are called “packages of weather” due to the effect on multiple weather variables simultaneously (Stenseth et al. 2003). The link between climatic oscillations and the corresponding ecosystem response are called teleconnections (Heffernan et al. 2014). Present results show that a significant part of the variability in interannual landings of European anchovy and European sardine stocks could have a relationship with the large-scale climate oscillations NAO, AO, and EA.

In summary, our results reveal the impact of short-term climatic oscillations on European anchovy and European sardine landings. Fishing is primarily an economic activity, and climate variability can also affect economic performance by driving changes in catch prices, due to the effect on supply. Finally, the planet is experiencing global warming. According to most forecasts, the climatic indices will become more and more extreme (for example Báez et al. 2021), and as has been highlighted in this study, the extreme values of the climatic oscillations in most models are those that have the greatest impact. Thereby, those climatic oscillations should be incorporated into fishery management, and future studies should focus on finding the mechanisms involved at the regional level. The bioeconomic models created allow for ecological interpretations, and therefore it is possible to link ecological management with economical implications. In this context, in agreement with Báez et al. (2021), input-based control measurements should be preferred in these highly variable and unpredictable situations.

## Supplementary Information

Below is the link to the electronic supplementary material.

Supplementary file1 (DOCX 15.5 KB)

Supplementary file2 (DOCX 14.4 MB)

Supplementary file3 (DOCX 14.2 MB)

Supplementary file4 (ZIP 75 KB)

## Data Availability

The datasets generated and/or analysed during the current study are available in the Sistema de Información Andaluz de Comercialización y Producción Pesquera of the Junta de Andalucía repository, http://www.juntadeandalucia.es/agriculturaypesca/idapes/servlet/FrontController.

## Code availability

All the R scripts will be accessible in the supplementary information.
