# Supplementary material for: Effect of climatic oscillations on small pelagic fisheries and its economic profit in the Gulf of Cadiz
Source: Int J Biometeorol. 2021 Nov 27;66(3):613–26. doi: 10.1007/s00484-021-02223-9 (PMC8850237; doi:10.1007/s00484-021-02223-9)
Supplement: Supplementary file 1 — Supplementary file1 (DOCX 15.5 KB) [file 484_2021_2223_MOESM1_ESM.docx]

**International Journal of Biometeorology**

**Effect of climatic oscillations on small pelagic fisheries and its economic profit in the Gulf of Cadiz**

Castro-Gutiérrez, J.^1*^, Cabrera-Castro, R.^1, 2^, Czerwinski, I. A.^1, 3^ and Báez, J. C.^4, 5^.

1. Departamento de Biología. Facultad de Ciencias del Mar y Ambientales, Universidad de Cádiz. Campus de Excelencia Internacional del Mar (CEIMAR). Avda. República Saharaui, s/n 11510 Puerto Real, Cadiz, Spain.

2. Instituto Universitario de Investigación Marina (INMAR). Campus de Excelencia Internacional del Mar (CEIMAR). Avda. República Saharaui, s/n 11510, Puerto Real, Cádiz, Spain.

3. Instituto Español de Oceanografía (IEO-CSIC), Centro Oceanográfico de Cadiz, Puerto Pesquero, Muelle de Levante, s/n, 11006 Cadiz, Spain.

4. Instituto Español de Oceanografía (IEO-CSIC), Centro Oceanográfico de Málaga, Puerto Pesquero de Fuengirola s/n, 29640 Fuengirola, Spain.

5. Instituto Iberoamericano de Desarrollo Sostenible, Universidad Autónoma de Chile, Temuco, Chile.

*** Corresponding author:** jairo.castrogutierrez@alum.uca.es; Tel.: +34 667 044 221; https://orcid.org/0000-0002-4466-3645

**Online Resource 1. Summary of the variables**

Table SS1. Summary of the variables used in this study. The letters "w" and "s" after the name of the climatic variables correspond to the winter and summer sub-variables, respectively. The number after the name of the climatic variables means the amount of lag (in years) used. All variables and sub-variables were squared (_sq) and cubed (_cb).

| **Climatic oscillations** | **Original variables** | **Transformed variables** | | | | | | |
| --- | --- | --- | --- | --- | --- | --- | --- | --- |
| **North Atlantic Oscillation (NAO)** | NAO | NAO_sq | NAO1 | NAO1_sq | NAO2 | NAO2_sq | NAO3 | NAO3_sq |
|  |  | NAO_cb |  | NAO1_cb |  | NAO2_cb |  | NAO3_cb |
|  | NAOw | NAOw_sq | NAOw1 | NAOw1_sq | NAOw2 | NAOw2_sq | NAOw3 | NAOw3_sq |
|  |  | NAOw_cb |  | NAOw1_cb |  | NAOw2_cb |  | NAOw3_cb |
|  | NAOs | NAOs_sq | NAOs1 | NAOs1_sq | NAOs2 | NAOs2_sq | NAOs3 | NAOs3_sq |
|  |  | NAOs_cb |  | NAOs1_cb |  | NAOs2_cb |  | NAOs3_cb |
| **Arctic Oscillation (AO)** | AO | AO_sq | AO1 | AO1_sq | AO2 | AO2_sq | AO3 | AO3_sq |
|  |  | AO_cb |  | AO1_cb |  | AO2_cb |  | AO3_cb |
|  | AOw | AOw_sq | AOw1 | AOw1_sq | AOw2 | AOw2_sq | AOw3 | AOw3_sq |
|  |  | AOw_cb |  | AOw1_cb |  | AOw2_cb |  | AOw3_cb |
|  | AOs | AOs_sq | AOs1 | AOs1_sq | AOs2 | AOs2_sq | AOs3 | AOs3_sq |
|  |  | AOs_cb |  | AOs1_cb |  | AOs2_cb |  | AOs3_cb |
| **East Atlantic Pattern (EA)** | EA | EA_sq | EA1 | EA1_sq | EA2 | EA2_sq | EA3 | EA3_sq |
|  |  | EA_cb |  | EA1_cb |  | EA2_cb |  | EA3_cb |
|  | EAw | EAw_sq | EAw1 | EAw1_sq | EAw2 | EAw2_sq | EAw3 | EAw3_sq |
|  |  | EAw_cb |  | EAw1_cb |  | EAw2_cb |  | EAw3_cb |
|  | EAs | EAs_sq | EAs1 | EAs1_sq | EAs2 | EAs2_sq | EAs3 | EAs3_sq |
|  |  | EAs_cb |  | EAs1_cb |  | EAs2_cb |  | EAs3_cb |
