# Supplementary material for: Effect of climatic oscillations on small pelagic fisheries and its economic profit in the Gulf of Cadiz
Source: Int J Biometeorol. 2021 Nov 27;66(3):613–26. doi: 10.1007/s00484-021-02223-9 (PMC8850237; doi:10.1007/s00484-021-02223-9)
Supplement: Supplementary file 2 — Supplementary file2 (DOCX 14.4 MB) [file 484_2021_2223_MOESM2_ESM.docx]

**International Journal of Biometeorology**

**Effect of climatic oscillations on small pelagic fisheries and its economic profit in the Gulf of Cadiz**

Castro-Gutiérrez, J.^1*^, Cabrera-Castro, R.^1, 2^, Czerwinski, I. A.^1, 3^ and Báez, J. C.^4, 5^.

1. Departamento de Biología. Facultad de Ciencias del Mar y Ambientales, Universidad de Cádiz. Campus de Excelencia Internacional del Mar (CEIMAR). Avda. República Saharaui, s/n 11510 Puerto Real, Cadiz, Spain.

2. Instituto Universitario de Investigación Marina (INMAR). Campus de Excelencia Internacional del Mar (CEIMAR). Avda. República Saharaui, s/n 11510, Puerto Real, Cádiz, Spain.

3. Instituto Español de Oceanografía (IEO-CSIC), Centro Oceanográfico de Cadiz, Puerto Pesquero, Muelle de Levante, s/n, 11006 Cadiz, Spain.

4. Instituto Español de Oceanografía (IEO-CSIC), Centro Oceanográfico de Málaga, Puerto Pesquero de Fuengirola s/n, 29640 Fuengirola, Spain.

5. Instituto Iberoamericano de Desarrollo Sostenible, Universidad Autónoma de Chile, Temuco, Chile.

*** Corresponding author:** jairo.castrogutierrez@alum.uca.es; Tel.: +34 667 044 221; https://orcid.org/0000-0002-4466-3645

**Online Resource 2. Model evaluation**

Multicollinearity between explanatory variables was checked through analysis of the variance inflation factor (VIF) (Fox and Monette, 1992). The goodness-of-fit of the GLMs was evaluated by generating another linear regression between observed and predicted values to obtain the coefficient of determination (R^2^) and thus compare the fit of the models. The R^2^ value describes the proportion of the total variance in the observed data that can be explained by the model. The Hosmer-Lemeshow test was used to test the goodness-of-fit of logistic regression models, while a confusion matrix was used to numerically evaluate the performance of the models. The confusion matrix consists of four elements (true positive, TP; false positive, FP; true negative, TN; and false negative, FN) and was used to obtain two specific assessment metrics:

(a) Accuracy: Percentage of positive and negative predictions that are correct, following the formulae:

$$Accuracy = \frac{TP + TN}{TP + TN + FP + FN}$$

(b) Precision: Percentage of positive predictions that are correct, following the formulae:

$$Precision = \frac{\mathrm{TP}}{TP + FP}$$

In the analysis of binary models, the receiver operator characteristic (ROC) curve is heavily used to show the performance of a model. The ROC curve is informative about the performance over a series of thresholds and can be summarised by the area under the curve (AUC), a single number (Muschelli, 2019). AUC represents the model's discriminative capacity by plotting the commission error (1 − specificity; false positives) on the horizontal axis vs. omission error (sensitivity; correctly identified positives) at the vertical axis. AUC ranges between 0.5–1, where 1 represents a perfect discrimination between presence and absence and 0.5 represents a random fit. AUC, as an evaluation metric, is threshold independent (Franklin, 2010).

Residual autocorrelation was assessed using autocorrelation function (ACF) and partial autocorrelation function (PACF) plots. Cook's distance was used to detect observations that strongly influence fitted values of the model. Influential values are those whose Cook distance is greater than 1 (Cook and Weisberg, 1982).

**References**

Cook RD, Weisberg S (1982) Residuals and Influence in Regression, Chapman and Hall, London.

Fox J, Monette G (1992) Generalized collinearity diagnostics. Journal of the American Statistical Association 87(417): 178-183. <https://doi.org/10.1080/01621459.1992.10475190>

Franklin J (2010) Mapping Species Distributions: Spatial Inference and Prediction. Cambridge University Press, NY, pp. 340.

Muschelli J (2019) ROC and AUC with a binary predictor: a potentially misleading metric. Journal of Classification 37(3): 696-708.
