# Supplementary material for: Effect of climatic oscillations on small pelagic fisheries and its economic profit in the Gulf of Cadiz
Source: Int J Biometeorol. 2021 Nov 27;66(3):613–26. doi: 10.1007/s00484-021-02223-9 (PMC8850237; doi:10.1007/s00484-021-02223-9)
Supplement: Supplementary file 3 — Supplementary file3 (DOCX 14.2 MB) [file 484_2021_2223_MOESM3_ESM.docx]

**International Journal of Biometeorology**

**Effect of climatic oscillations on small pelagic fisheries and its economic profit in the Gulf of Cadiz**

Castro-Gutiérrez, J.^1*^, Cabrera-Castro, R.^1, 2^, Czerwinski, I. A.^1, 3^ and Báez, J. C.^4, 5^.

1. Departamento de Biología. Facultad de Ciencias del Mar y Ambientales, Universidad de Cádiz. Campus de Excelencia Internacional del Mar (CEIMAR). Avda. República Saharaui, s/n 11510 Puerto Real, Cadiz, Spain.

2. Instituto Universitario de Investigación Marina (INMAR). Campus de Excelencia Internacional del Mar (CEIMAR). Avda. República Saharaui, s/n 11510, Puerto Real, Cádiz, Spain.

3. Instituto Español de Oceanografía (IEO-CSIC), Centro Oceanográfico de Cadiz, Puerto Pesquero, Muelle de Levante, s/n, 11006 Cadiz, Spain.

4. Instituto Español de Oceanografía (IEO-CSIC), Centro Oceanográfico de Málaga, Puerto Pesquero de Fuengirola s/n, 29640 Fuengirola, Spain.

5. Instituto Iberoamericano de Desarrollo Sostenible, Universidad Autónoma de Chile, Temuco, Chile.

*** Corresponding author:** jairo.castrogutierrez@alum.uca.es; Tel.: +34 667 044 221; https://orcid.org/0000-0002-4466-3645

**Online Resource 3. Preliminary exploration results.**

**European anchovy**

Boxplot and dotchart results (Figure SS1) showed the three highest landings as outliers. These values correspond to the years 1997, 1998, and 1999.


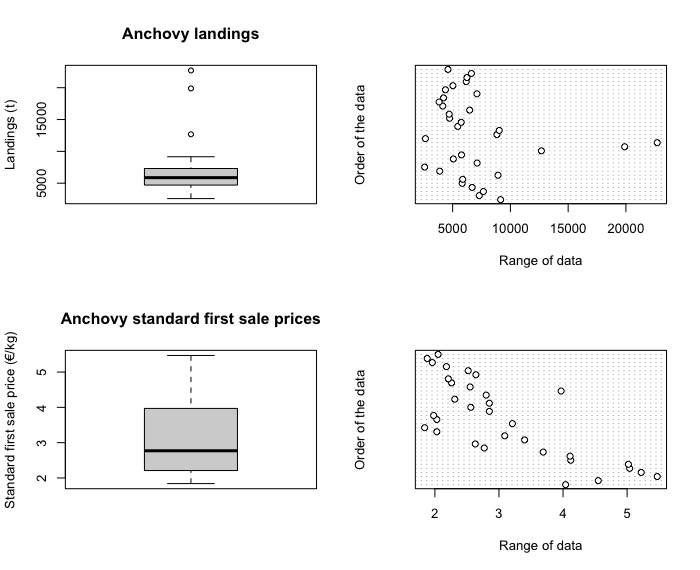


**Fig SS1.** Boxplots (left) and dotcharts (right) of European anchovy landings and standard first sale price variables

European anchovy landings reached the maximum value in 1999 with 22 709.66 t and the minimum value in 1993 with 2 577.92 t. Five main periods were identified by the changepoint analysis (Figure SS2). European anchovy standard first sale price reached the maximum value in 1987 with 5.47 €/kg and a minimum value in 1999 with 1.84 €/kg. Six main periods were identified by the changepoint analysis (Figure SS2). Table SS1 shows the numerical results of the changepoint analysis. The third European anchovy landing period had a higher mean than the rest and the species presented a high variance in all periods. The highest standard first sale price variances were found in periods 1 and 4. The European anchovy was the only species who’s standard first sale price showed a significative trend (S = -310; p<0.05) in the Mann-Kendall test.


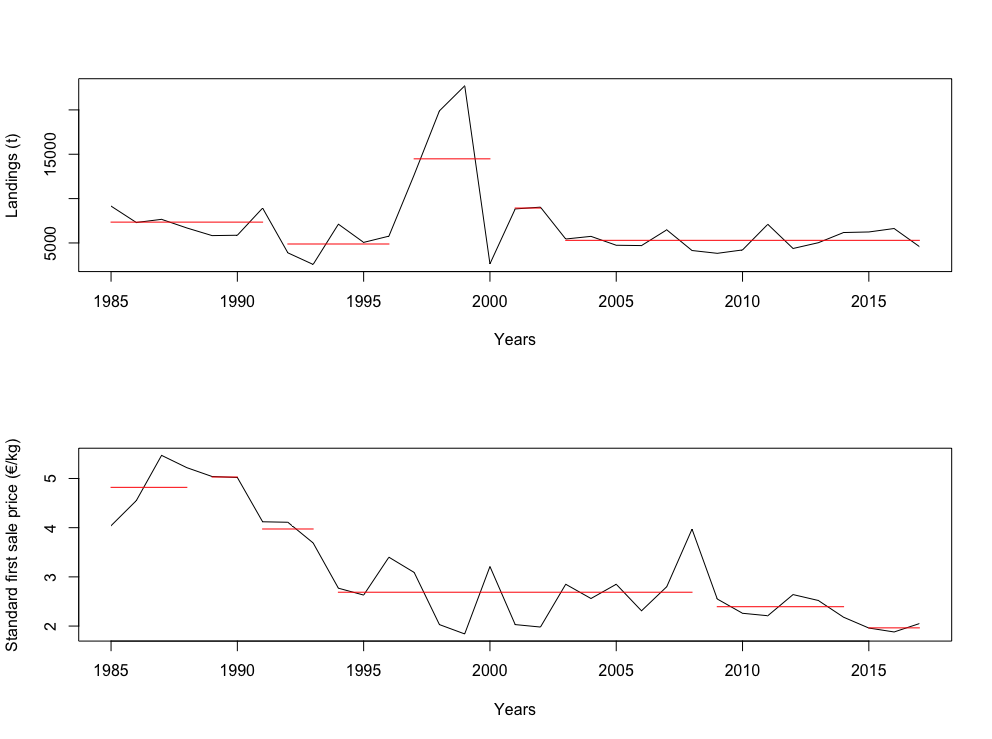


**Fig SS2** Time series from 1985 to 2017 of European anchovy landings (upper plot) and standard first sale price (bottom plot). Red lines represent the main periods identified by the changepoint analysis.

**Table SS1**. Numerical results of European anchovy changepoint analysis

| **Variable** |  | **Main periods** | | | | | |
| --- | --- | --- | --- | --- | --- | --- | --- |
|  |  | **1** | **2** | **3** | **4** | **5** | **6** |
| **Landings (t)** | **Years** | 1985 - 1991 | 1992 - 1996 | 1997 - 2000 | 2001 - 2002 | 2003 - 2017 |  |
|  | **Mean** | 7348.46 | 4882.29 | 14482.31 | 8935.66 | 5296.18 |  |
|  | **Variance** | 1541718.27 | 2423980.90 | 60069548.33 | 10147.34 | 1001727.35 |  |
| **Standard first sale price (€/kg)** | **Years** | 1985 - 1988 | 1989 - 1990 | 1991 - 1993 | 1994 - 2008 | 2009 - 2014 | 2015 - 2017 |
|  | **Mean** | 4.82 | 5.03 | 3.97 | 2.68 | 2.39 | 1.96 |
|  | **Variance** | 0.31 | 0.00 | 0.04 | 0.33 | 0.03 | 0.00 |

Autocorrelation plots (Figure SS3) showed temporal correlation in European anchovy standard first sale prices and no apparent visual cyclicity. Collinear pairs of explanatory variables are shown in Table SS2. These pairs were not introduced together in the models.


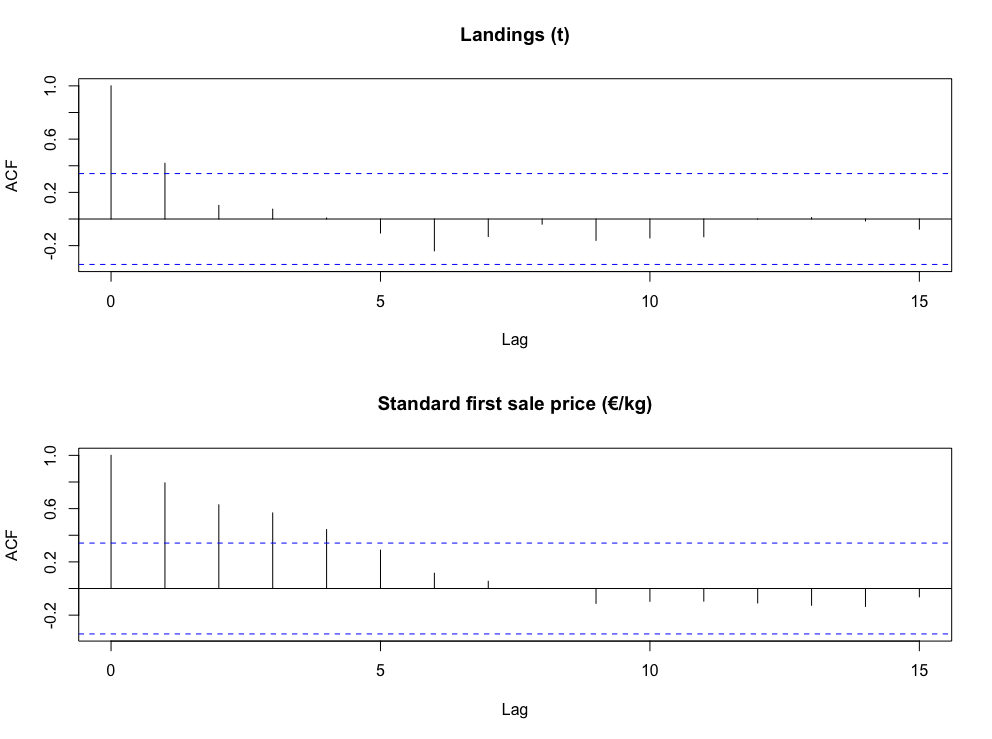


**Fig SS3** Autocorrelation plots for European anchovy data using 15 years of lag

**Table SS2**. Pearson correlation coefficients together with the pairs of collinear variables. Key: NAO, North Atlantic Oscillation; AO, Arctic Oscillation; EA, East Atlantic pattern. The letters "w" and "s" after the name of the climate variable correspond to the winter and summer sub-variable, respectively. The number after the name of the climate variable means the amount of lag (in years) used.

| **Pairs of explanatory variables** | | **Pearson's r coefficient** |
| --- | --- | --- |
| NAO | AO | 0,772 *** |
| NAO1 | AO1 | 0,754 *** |
| NAO2 | AO2 | 0,749 *** |
| NAO3 | AO3 | 0,790 *** |
| NAOw | AOw | 0,805 *** |
| NAOw1 | AOw1 | 0,806 *** |
| NAOw2 | AOw2 | 0,802 *** |
| NAOw3 | AOw3 | 0,803 *** |
| NAOs2 | AOs2 | 0,753 *** |
| NAOs2 | EAs1 | -0,713 *** |
| NAOs3 | AOs3 | 0,738 *** |
| EA | EAs | 0,784 *** |
| EA1 | EAs1 | 0,802 *** |
| EA2 | EAs2 | 0,740 *** |
| EA3 | EAs3 | 0,739 *** |

Asterisks (***) describe a significance level p<0.001.

**European sardine**

The boxplots and dotcharts (Figure SS4) showed the four highest standard first sale prices as outliers. These values correspond to the years 2012, 2014, 2015, and 2017.


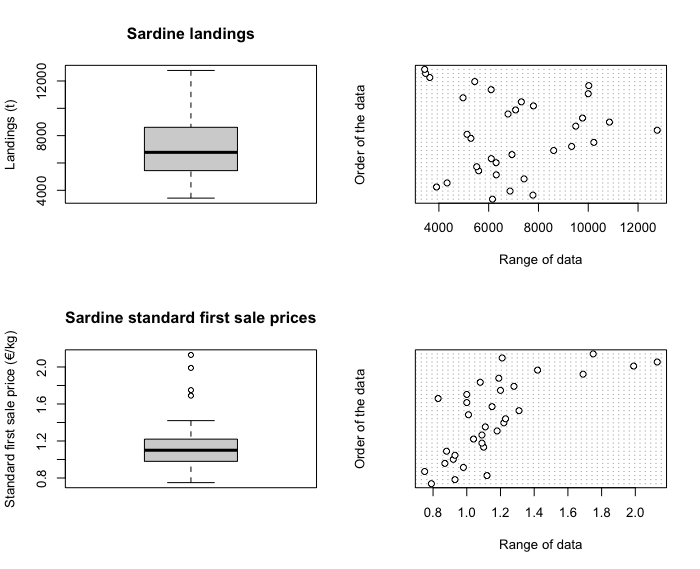


**Fig SS4** Boxplots (left) and dotcharts (right) of European sardine landings and standard first sale price variables.

European sardine landings reached the maximum value in 2002 with 12 770.88 t and the minimum value in 2017 with 3 427,86 t. Four main periods were identified by the changepoint analysis (Figure SS5). The highest mean was found in the third period and a high variance was found in all periods. European sardine standard first sale price reached its maximum value in 2015 at 2.07 €/kg and its minimum in 1988 at 0.32 €/kg. Five periods were identified by the changepoint analysis (Figure SS5). The mean was increasing throughout the periods and no high variance was found. Table SS3 shows the numerical results of the changepoint analysis. Mann-Kendall trend test showed a significative trend in European sardine total annual value (S = 316; p<0.05) and standard first sale prices (S = 295; p<0.05).


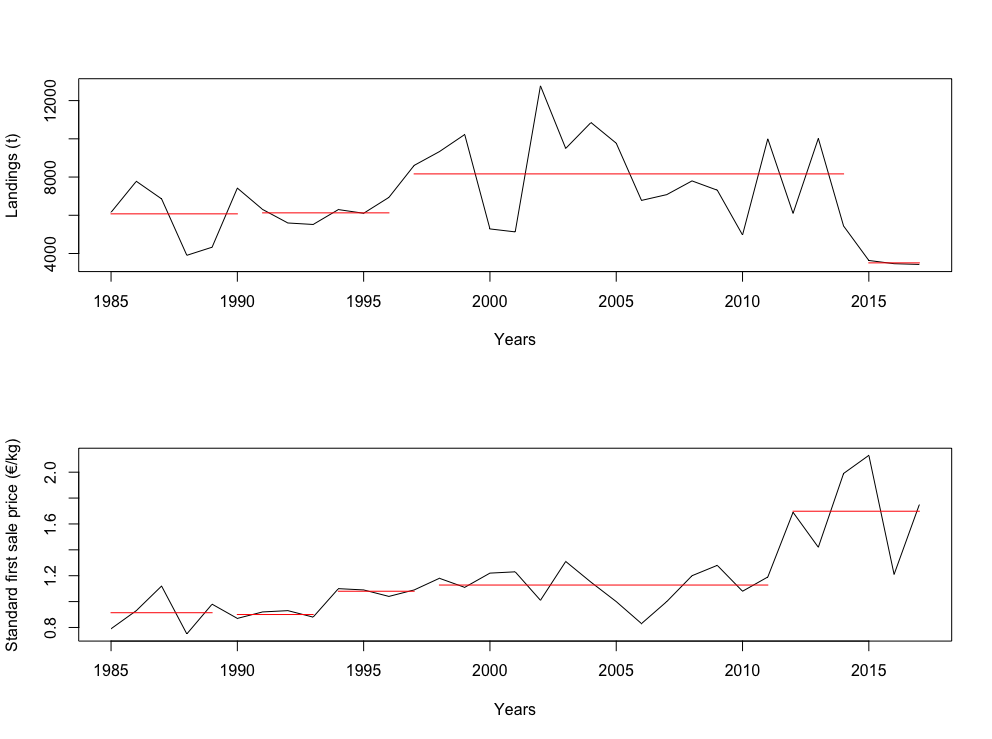


**Fig SS5** Time series from 1985 to 2017 of European sardine landings (upper plot) and standard first sale price (bottom plot). Red lines represent the main periods identified by the changepoint analysis.

**Table SS3**. Numerical results of European sardine changepoint analysis.

| **Variable** |  | **Main periods** | | | | |
| --- | --- | --- | --- | --- | --- | --- |
|  |  | **1** | **2** | **3** | **4** | **5** |
| **Landings (t)** | **Years** | 1985 - 1990 | 1992 - 1996 | 1997 - 2014 | 2015 - 2017 |  |
|  | **Mean** | 6074.37 | 6126.52 | 8165.03 | 3509.89 |  |
|  | **Variance** | 2181412.85 | 227718.62 | 4930903.33 | 8087.58 |  |
| **Standard first sale price (€/kg)** | **Years** | 1985 - 1989 | 1990 - 1993 | 1994 - 1997 | 1998 - 2011 | 2012 - 2017 |
|  | **Mean** | 0.91 | 0.90 | 1.08 | 1.13 | 1.70 |
|  | **Variance** | 0.02 | 0.00 | 0.00 | 0.02 | 0.10 |

Autocorrelation plots (Figure SS6) showed temporal correlation in European sardine standard first sale prices and no apparent visual cyclicity.


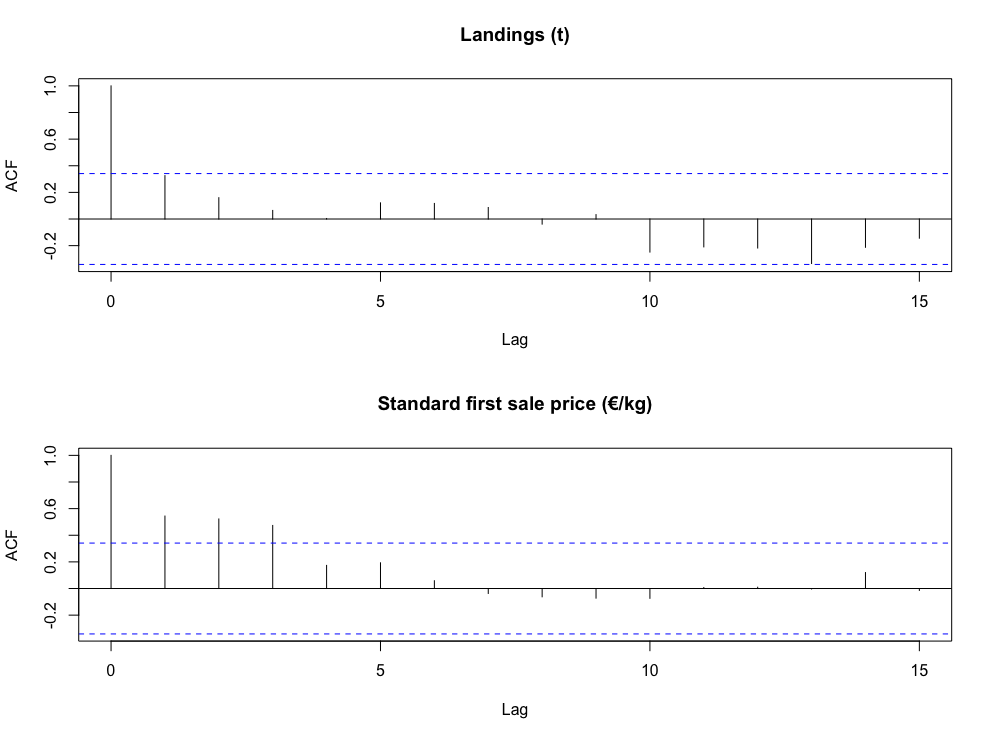


**Fig SS6** Autocorrelation plots for European sardine data using 15 years of lag.
